# rSW-seq: Algorithm for detection of copy number alterations in deep sequencing data

**DOI:** 10.1186/1471-2105-11-432

**Published:** 2010-08-18

**Authors:** Tae-Min Kim, Lovelace J Luquette, Ruibin Xi, Peter J Park

**Affiliations:** 1 Center for Biomedical Informatics, Harvard Medical School, 10 Shattuck St, Boston, Massachusetts 02115, USA; 2 Department of Medicine, Brigham and Women's Hospital, 77 Avenue Louis Pasteur, Boston, Massachusetts 02115, USA; 3 Harvard-MIT Health Sciences and Technology Informatics Program at Children's Hospital, 300 Longwood Ave., Boston, Massachusetts 02115, USA

## Abstract

**Background:**

Recent advances in sequencing technologies have enabled generation of large-scale genome sequencing data. These data can be used to characterize a variety of genomic features, including the DNA copy number profile of a cancer genome. A robust and reliable method for screening chromosomal alterations would allow a detailed characterization of the cancer genome with unprecedented accuracy.

**Results:**

We develop a method for identification of copy number alterations in a tumor genome compared to its matched control, based on application of Smith-Waterman algorithm to single-end sequencing data. In a performance test with simulated data, our algorithm shows >90% sensitivity and >90% precision in detecting a single copy number change that contains approximately 500 reads for the normal sample. With 100-bp reads, this corresponds to a ~50 kb region for 1X genome coverage of the human genome. We further refine the algorithm to develop rSW-seq, (recursive Smith-Waterman-seq) to identify alterations in a complex configuration, which are commonly observed in the human cancer genome. To validate our approach, we compare our algorithm with an existing algorithm using simulated and publicly available datasets. We also compare the sequencing-based profiles to microarray-based results.

**Conclusion:**

We propose rSW-seq as an efficient method for detecting copy number changes in the tumor genome.

## Background

Human solid tumors harbor various types of chromosomal alterations, many of which play a role in the initiation and progression of the disease [1,2]. As a major category of chromosomal alterations, DNA copy number alterations (CNAs) that represent chromosomal gains or losses have been extensively investigated in cancer research. Many CNAs can affect the function or structure of cancer-related genes and are associated with causative molecular mechanisms in carcinogenesis. Thus, a comprehensive catalogue of CNAs in a given tumor type is an important step in understanding the underlying carcinogenic mechanisms and in highlighting potential biomarkers with diagnostic or therapeutic implications.

In recent years, high-resolution array Comparative Genomic Hybridization (array-CGH) has become a standard platform for identification of CNAs in a genome-scale and great progress has been made in profiling of cancer-related chromosomal alterations with improved spatial resolution [3,4]. In spite of the many successes, array-CGH has several limitations inherent in hybridization-based techniques, such as noise due to cross-hybridization between probe and target sequences as well as a limited and nonlinear dynamic range. In addition, the resolution and genome coverage of an array-CGH platform are dependent on a fixed set of probes, making it difficult to identify novel alterations below a given size [5].

The first use of sequencing data in genome-wide identification of CNAs was digital karyotyping [6]. Its utility, however, was limited by the cost of conventional Sanger sequencing method. Fortunately, the recent arrival of next-generation sequencing technology has altered the situation dramatically. This technology allows large-scale sequencing data to be generated with significantly lower cost and higher throughput [7,8]. Although the advantage of this sequencing technology has been already shown in a wide spectrum of genomic applications [9,10], more accurate and robust methods are needed for identification of copy number alterations for the large amount of whole-genome sequencing data that will be generated in the near future.

There are two classes of methods for copy number assessment, both based on the assumption that the local density of sequenced reads is proportional to the copy number. The first is to estimate copy number in a single sample, typically to identify copy number variation (CNV) of a non- diseased individual (although there is no consensus, CNV often refers to all alterations, both germline and somatic, in contrast to CNA for somatic alterations). In this case, a 'read depth' can be measured for non-overlapping genomic windows and used to identify CNVs with respect to a reference genome. This strategy has been addressed elsewhere [11,12], but it is complicated by other factors, such as local GC content, that affect the read density significantly. The second class of methods is to estimate copy number in one genome compared to its control, typically in a disease tissue versus a normal tissue from the same individual. This has the advantage of controlling for patient-specific CNVs, thus shifting the focus to somatic alterations. The disadvantage is that the number of experiments required is doubled. In this study, we propose a method for the second case in which sequencing reads are available for two matched genomes. We focus on cancer genomes here, but it can be applied to comparison of any two genomes.

With the sequencing data from the tumor and its paired normal genomes, CNAs are characterized by a disproportionately higher number of tumor reads (copy number gains) or normal reads (losses). Theoretically, the spatial resolution and the dynamic range of the detected copy number changes are limited only by the sequencing depth, unlike in the fixed resolution of the array-CGH platforms. The approach we take is based on a modification of the Smith-Waterman algorithm [13]. This idea was previously proposed for analysis of array-CGH [14]. Here, we adapt it for sequencing data and introduce further improvements. In simulation tests, our method is able to detect even a single copy change in a region with high sensitivity and precision. To identify a set of alterations in a multilayered configuration with different copy numbers, we propose a recursive version of the method called rSW-seq (recursive Smith-Waterman-seq). We compare our method with a previously published algorithm SegSeq [15], using simulated and publicly available sequencing data.

## Results and Discussion

We start with sequencing datasets obtained from a tumor and its matched normal genomes. Under the null hypothesis of no copy number difference, a genomic segment would have an expected read ratio close to (total number of tumor reads)/(total number of normal reads). A read ratio showing substantial deviation from this expected ratio would be indicative of copy number alterations. One simple approach is to use a moving-window to generate read ratios along the genome, analogous to the probe-specific intensity ratios in conventional array-CGH profiles. Then, a known segmentation algorithm designed for array-CGH data can be applied [16,17]. However, this is computationally expensive for the sequencing data and does not take full advantage of the data. Alternatively, one can use the density of reads to determine whether the ratio is significantly different from 1 for each window based, for instance, on the normal or Poisson distribution. Then the neighboring windows with significant amplification or deletion can be joined together. A sliding window of fixed width is simplest, but because this results in unstable ratios in regions with small read counts, a window may be defined by a fixed number of reads in the normal sample. Non-overlapping windows are typically used, as this makes tests in adjacent regions independent and reduces the computational burden; but overlapping windows can be also used, especially to generate a smoothed profile. SegSeq, a recently proposed sequencing-based algorithm, utilizes windows defined by a predefined number of normal reads to detect breakpoints between CNAs [15]. A major disadvantage of window-based approaches, however, is that the window size must be determined a *priori*, and that the overall performance of the algorithm is influenced strongly by that value. For example, a larger window size enhances the confidence level of CNAs identified [18], but too large a window sacrifices spatial resolution. The method we propose below avoids having to define a window.

### Description of the algorithm

**Figure 1:**
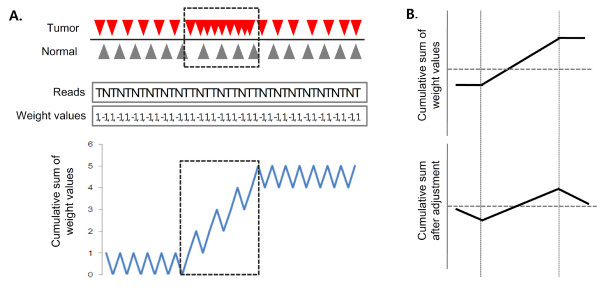
**A schematic of the algorithm**. **(A) **The sequencing reads for tumor (red triangles) and matched normal genomes (black triangles) are shown. The reads are ordered according to their chromosomal location and converted into a one-dimensional array for pattern detection. Tumor (T) and normal (N) reads are given the weight values *W_T _*and *W_N_*, respectively (in this example, +1 and -1 for simplicity). The cumulative sum of weight values shows an upward slope (indicated by the box) for a region of local copy number gain (prevalence of tumor reads over normal reads). **(B) **The upward slope in the cumulative sum and the flanking flat lines correspond to a local copy number gain and regions of no copy number change, respectively (top). For improved performance, the threshold *t *is subtracted from weight values to give a negative slope to regions of no copy number differences while maintaining the positive slope for the copy number gain (bottom).

The sequencing reads from tumor and matched normal genome are combined and sorted in a non-decreasing order according to their genomic positions (Figure 1A). The reads from tumor and normal genomes are distinguished and assigned different weight values of *W_T _*and *W_N_*, respectively. When the number of reads for the tumor and normal samples (*N_T _*and *N_N_*, respectively) are equal, they are assigned equal weight but with different signs (e.g., *W_T _*= 1 and *W_N _*= -1). Otherwise, (*N_T _*≠ *N_N_*), the weights for tumor and normal reads are set given the *N_T _*and *N_N _*(e.g, *W_T _*= 2 × *N_N_*/(*N_T _*+ *N_N_*) and *W_N _*= -2 × *N_T_*/(*N_T _*+ *N_N_*)) This equalizes the total sum of *W_T _*and $-W_{N}\left(\sum_{1}^{N_{T}}W_{T}=-\sum_{1}^{N_{N}}W_{N}\right)$, making the sum of all *W_T _*and *W_N _*to be zero. Thus, the sequencing data from tumor and matched normal genome is converted into a one-dimensional vector of *W_T _*and *W_N_*, amenable to an algorithm for pattern detection.

The main idea of our method is that a large local positive or negative cumulative sum in this vector of weight values indicates a local copy number gain or loss, respectively. As shown in Figure 1A, the local copy number gain (prevalence of tumor reads over normal reads) results in an upward slope of the cumulative sum. To identify the alterations and to map the boundaries accurately and rapidly in this cumulative sum profile, we propose to use the Smith-Waterman algorithm. This algorithm was originally developed to determine highly conserved, consecutive nucleotides in the local sequence alignment problem [13]. The use of the Smith-Waterman algorithm for copy number analysis was previously proposed by Price *et al. *[14] for array-CGH data in their SW-ARRAY algorithm. We have found that this algorithm is also suitable for copy number estimation from sequencing data with appropriate modifications. Thus, in this work, we have adopted the modified Smith-Waterman algorithm to map the copy number changes.

In this method, the tumor-specific copy number gains and losses are identified separately. Assume that the reads on a chromosome are *r*_1 _= (*W*_1_,*s*_1_),...,*r_n _*= (*W_n_, s_n_*), where *W_j _*and *s_j _*are the weight and the mapped location associated with the read *r_j_*, respectively. Since the short reads are ordered, we have *s*_1 _≤ *s*_2 _≤⋯≤ *s_n _*For copy number gain, the algorithm searches for the segment [*s_l_, s_m_*] such that the partial cumulative sum $S(l,m)=\sum_{j=l}^{m}W_{j}$ is maximized. Then we iterate until no more alternation can be found.

Specifically, let *l*_1 _= 1 and $l_{k+1}=\min\{l\geq l_{k}:S(l_{k},l)=\sum_{j=l_{k}}^{l}W_{j}<0\}+1$, i.e., *l*_*k*+1 _-1is the first index after *l_k _*such that. S(*l_k_, l*) < 0 (*l *>*l_k_*) Suppose that after certain *k *≥ 1, we have *S*(*l_k_, l*) ≥ 0 for all *l *≥ *l_k_*. Denote *l*_*k*+1 _= *n *+ 1. We then let *mk *= argmax {*S*(*l_k_, m*), *m *∈ [*l*_*k*_,*l*_*k*+1_]}, i.e., *m_k _*is the index between *l_k _*and *l*_k+1 _such that *S*(*l_k_, m*) is maximized. Then, the partial cumulative sums *S*(*l_k_, m_k_*) will be maximized at some *k*_0 _∈ {1,...,*K*}. One can show that the segment $\left[S_{l_{k_{0}}},S_{m_{k_{0}}}\right]$ is the maximum segment [*s_l_, s_m_*] that maximizes the partial cumulative sum $S(l,m)=\sum_{j=l}^{m}W_{j}$ over all 1 ≤ *l *≤ *m *≤ *n *(see Methods). The algorithm rSW-seq just iteratively searches for *l_k _*and *m_k_*, starting from *l*_1 _= 1. Once the maximum segment $\left[S_{l_{k_{0}}},S_{m_{k_{0}}}\right]$ is identified, the region will be reported as a copy gain region if $S\left(S_{l_{k_{0}}},S_{m_{k_{0}}}\right)>0$. Then, the algorithm will mask this region, i.e., setting the weights *W_j_*of the reads in this region to be zero, and search for the next copy gain region until no further copy gain region can be identified. For copy number losses, the same method can be applied to the original array of weight values with the signs inverted for *W_T _*and *W_N_*. The pseudo-code for detecting positive-scoring segment is available in Methods. In practice, one does not scan the whole chromosome again for the next region of interest; instead, a ranked list of candidates [*s_l_, s_m_*] is kept and only the neighborhood of the identified variant is scanned again.

In Figure 1, the cumulative sum *S *should be close to zero in the regions of no copy number changes. However, a noisy distribution of reads might lead to a fluctuating pattern of local *S *and increase false positives in the selection of positive-scoring segments. To make the algorithm robust to noise, we subtract a predefined threshold level *t *from the weight values *W_T _*and *W_N _*globally. This adjustment gives a negative slope to regions with no copy number changes in the cumulative sum plot while maintaining the positive slope of the copy number gains (Figure 1B). This preprocessing helps to minimize the false positives without losing accuracy in mapping the boundaries of true copy number alterations. This point is illustrated with an example in the next section.

### Simulation tests

To measure the performance of the algorithm, we generated a set of 100 Mb artificial chromosomes on which 1 million random reads are mapped (See Methods for details on simulated data). The dependency of the algorithm on different sequencing depths is discussed later. We assume that the same numbers of virtual reads (half million reads each) are derived from the tumor and normal genomes. The tumor reads are positioned to generate regions of copy number ratios 3/2 or 1/2, corresponding to a single copy number of gain or loss, respectively. The single copy alterations were selected for the performance test since they represent the minimal ratio difference between tumor and normal reads, making them the most difficult to find. Different alteration sizes (10 kb to 1 Mb in 8 scales) were simulated with 100 artificial chromosomes for each size category.

**Figure 2:**
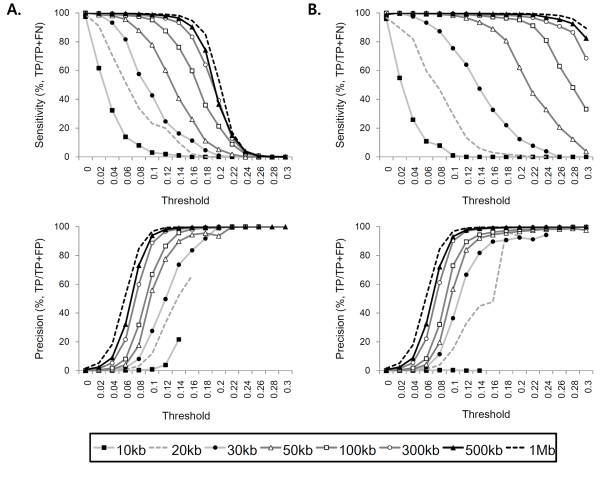
**The performance of the algorithm in simulation tests**. **(A) **The algorithm was tested for 16 different *t *values (0 - 0.3; *x*-axis) in detecting single copy gains with different sizes (10 kb to 1 Mb). The regions identified by the algorithm were compared with the predefined alterations. The performance was measured in terms of sensitivity (%; top panel) and precision (%; bottom panel). Each dot in the plot represents the average of 100 simulation tests. **(B) **Sensitivity (top) and precision (bottom) levels in detecting single copy losses.

First, we tested the algorithm for a wide range of *t *threshold values (16 levels, from 0 to 0.3 stepping at 0.02) and compared the identified candidate CNAs with the predefined alterations. The performance of the algorithm at different *t *levels was measured in terms of sensitivity (%; TP/TP + FN) and precision (%; TP/TP + FP) (Figure 2). We selected these measures to reflect two critical aspects of the algorithm's performance: (1) what percentage of known (simulated) alterations is correctly identified by the algorithm (sensitivity) and (2) what percentage of identified alterations by the algorithm are true positives (precision). Specificity, the percentage of non-altered regions correctly identified as such, is not as meaningful in this context because the non-altered regions comprise a very large fraction of the genome and specificity becomes less sensitive. Without any adjustment (*t *= 0), single copy gains and losses larger than 20 kb were identified with >90% sensitivity but the precision level was very low, indicating a high rate of false positives. With different *t *levels, a clear trade-off between sensitivity and precision was observed, as the increase in threshold improves precision at the expense of sensitivity. A balanced performance was obtained at *t *level around 0.1 (for single copy gains) and 0.16 (losses), respectively. At these *t *levels (*t_gain _*= 0.1 and *t_loss _*= 0.16), the algorithm achieved >90% sensitivity and >60% precision in detecting 100 kb single copy gains and >80% sensitivity and >80% precision levels for 50-kb single copy losses. For single copy gains, the smaller threshold values (0 <*t_gain _*< 0.1) are not sufficient in filtering out false positives and results in low precision; higher values (0.1 <*t_gain _*< 0.2), on the other hand, are associated with low sensitivity level. We note that the optimal threshold values found here are about half of the threshold values that make the local *S *of single copy gains and losses zero (*t *= 0.2 and *t *= 0.33, respectively). For example, consider a single copy gain with *n_t _*(tumor reads) and *n_n _*(normal reads) with read ratio (*n_t_/n_n_*) of 3/2. The *t *value that makes the sum of weight values to be zero can be calculated by an equation: *W_T _*× *n_t _*+ *W_N _*× *n_n _*-*t *× (*n_t _*+ *n_n_*) = 0. If *W_T _*= 1 and *W_N _*= -1 (*N_T _*= *N_N_*), the *t *is 0.2, the half of which is the empirically determined optimal *t_gain_*. For real data sets, this is a reasonable way to determine the initial value of *t*.

**Figure 3:**
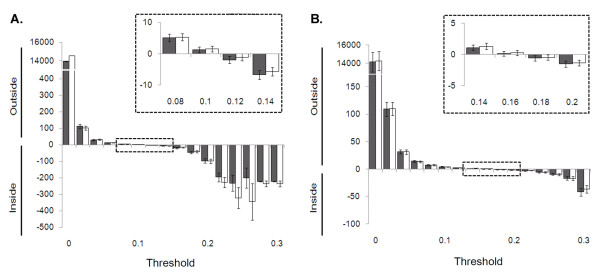
**Accuracy in mapping boundaries**. **(A) **The differences between observed and known boundaries of alterations are measured for different values of the threshold *t*. The distance (kb) was measured separately for the start (grey, closed) and end points (open) between the observed and predefined alterations. The relative location of mapped boundaries are divided into those located inside and outside of the predefined alterations. The inset magnifies the section for *t_gain _*of 0.08 to 0.14. The error bar shows the 95% confidence interval. **(B) **The measurement is repeated for single copy loss.

We further measured the effect of different *t *levels in the accuracy of boundary mapping (Figure 3). Both for the single copy gains and losses, the boundaries of observed alterations detected at lower *t *level tend to fall outside the predefined boundaries, while the opposite is true for higher *t *levels. In case of single copy gains, *t_gain _*= 0.1 also showed the highest accuracy in boundary mapping: 1.3 ± 0.8 kb and 1.5 ± 0.9 kb for start and end boundaries, respectively, with little dependence on the alteration size. For single copy losses, the accuracy range of 0.2 ± 0.4 kb and 0.3 ± 0.4 kb for start and end boundaries was observed at *t_loss _*of 0.16.

Because this algorithm involves scanning along the chromosomes, it may not give the same results when scanned in different directions. To check whether our method is robust with respect to scanning orientation, we applied the method in both directions at *t_gain _*of 0.1 and *t_loss _*of 0.16. Among the observed gains identified by left-to-right scanning, 88.6% were recovered with the exactly the same boundaries as by right-to-left scanning. This coincident rate for boundary mapping was much higher when considering only true positives (96.7%). In case of losses, most of the observed losses (99.8%) showed matching boundaries in both scanning directions.

The SW-score, which we define to be the local sum of *W_T _*and *W_N _*in an identified segment, can be used to rank identified regions. But this is biased toward a larger segment, which has a higher probability of generating a high score. Thus, we also introduce another measure of significance for each segment as an alternative or additional filter: the probability of finding the observed or more extreme distribution of tumor and normal reads in the identified region given the total number of tumor and normal reads. This can be done by assuming that the read density follows the Poisson or normal distribution. We adapt a statistical method previously described for differential analysis of sequencing tags based on the Poisson distribution [19] (see Methods). To see the effects of the additional screening, the alterations identified at *t_gain _*of 0.1 and *t_loss _*of 0.16 were filtered by their SW-scores (11 scales from 50 to 150) or significance levels (11 scales from 10^-5 ^to 10^-15^). The use of stringent cutoffs in both measures tends to increase precision when detecting small alterations while maintaining the sensitivity levels of large alterations (see Additional file 1: Figure S1). In detecting single copy number gains, for instance, the use of score threshold of 80 or significance of 10^-8 ^was optimal, showing >90% sensitivity and >90% precision in detecting 100 kb copy number gains. The similar performance level was observed in detecting 100 kb single copy number losses at the same significance cutoff (see Additional file 1: Figure S2).

**Figure 4:**
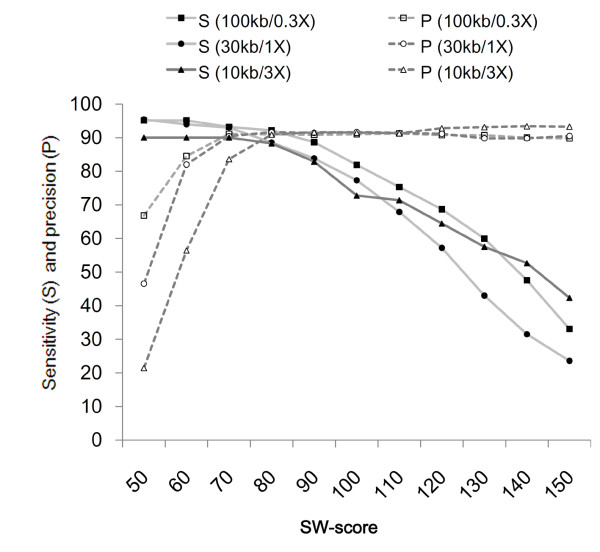
**Performance of the algorithm at different genome coverage**. Performance in identification of different size alterations was measured at different coverage levels. Besides the 100 kb alterations simulated at 0.36× coverage, the 1× and 3× coverage levels were simulated by putting 3 million and 10 million reads on 100 Mb, respectively. Sensitivity (S) and precision (P) were measured at different SW-score cutoffs.

Because the algorithm is dependent on relative tag density only, we expect that the regions with similar read numbers can be identified at a similar performance level regardless of their physical length. To test this, we simulated 30 kb and 10 kb single copy gains with 3 million and 10 million virtual reads in 100 Mb artificial chromosomes (Figure 4). The SW-score cutoff 80 gave consistent performance level (>90% sensitivity and >90% precision) for the simulated alterations that are expected to have approximately 500 reads for the normal sample.

**Figure 5:**
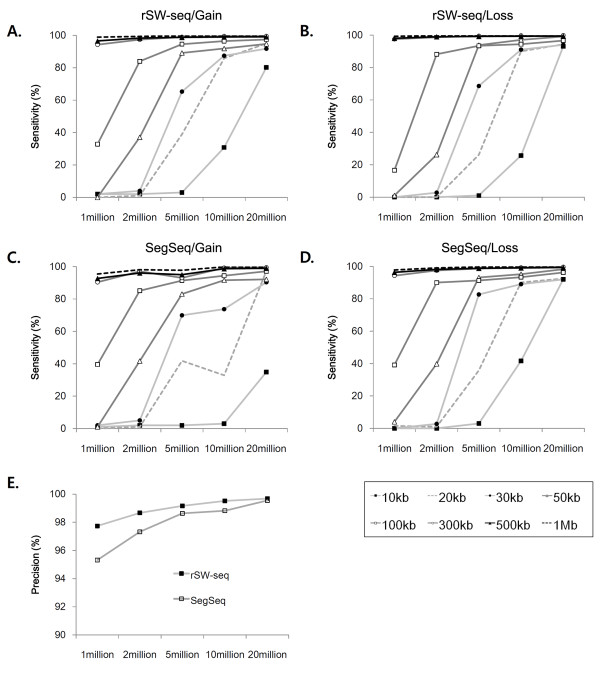
**Simulation tests on rSW-seq and SegSeq**. Different sizes (10 kb to 1 Mb) of single copy gains and losses were simulated on human chromosome 1. A hundred test chromosomes were simulated at varying sequencing depths (1 to 20 million reads). The sensitivity in detecting simulated alterations by rSW-seq is shown for single copy gain **(A) **and loss **(B)**. The same simulation sequencing data was also analyzed by SegSeq, which show similar sensitivity in detecting copy number gain **(C) **and loss **(D)**. The precision levels are shown for rSW-seq and SegSeq in **(E)**.

To further investigate dependency on different sequencing depth and to compare the results with SegSeq [15], we performed simulation tests that accounts for read mappability. Different sizes (10 kb - 1 Mb; 8 scales) of single copy gains and losses were simulated on human chromosome 1 (see Methods), in which random 36 bp reads were selected with varying sequencing depth (1 - 20 million reads) and aligned back to the genome. In this simulation, both algorithms show comparable sensitivity level with each other in detecting various sized alterations (Figure 5). The sensitivity level is dependent upon the alteration size and sequencing depth for both algorithms, e.g., rSW-seq and SegSeq both showed >90% of sensitivity at detecting 50 kb alterations with 5 million reads in simulated chromosome (~250 Mb). With low sequencing depth (<10 million reads in ~250 Mb chromosome), rSW-seq showed improved precision, indicative of low false positive rates compared to SegSeq (Figure 5E).

### Complex alterations and recursive SW-seq (rSW-seq)

Simulations of a single, isolated alteration in a chromosome does not fully represent the complexity of alterations commonly observed in a real cancer genome. For example, the high amplifications or homozygous deletions of well-known cancer-related genes such as *EGFR *and *CDKN2A *frequently occur within low-level chromosomal gains or losses, rather than in isolation. A simple chromosomal scan might miss such embedded high copy number changes, which frequently harbor important cancer-related genes. To distinguish these focal amplifications, the algorithm described above can be applied in a recursive manner by exploiting the fact that focal amplification is a relative copy number gain with respect to the single copy gain background.

Thus, using the single copy gain as a template, the recursive SW-seq (rSW-seq) can identify a focal, high-level amplification.

**Figure 6:**
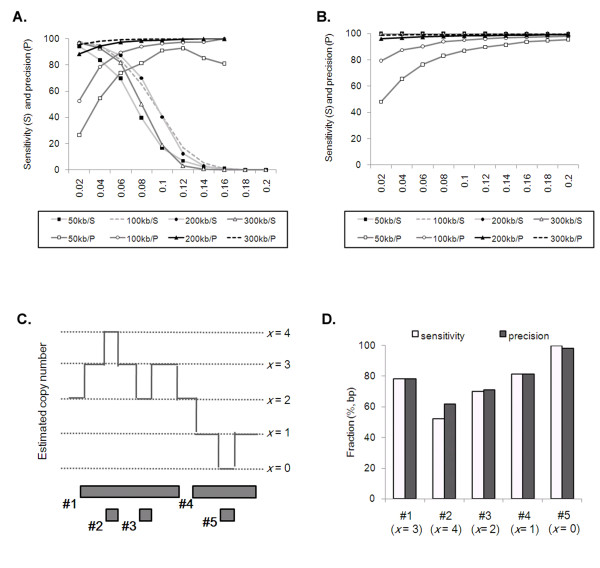
**Simulation tests for complex alterations and performance of rSW-seq**. **(A) **The performance in identification of focal, two copy number changes (4 copies) of various sizes (50 kb--300 kb) from 1 Mb single copy gains. The sensitivity (S) and precision (P) are shown for four size categories at different *t *levels. (**B**) The homozygous deletions (zero copy) nested in single copy losses were similarly tested. **(C) **Five alterations with different copy numbers (0 ~ 4 copies) are positioned in a complex configuration. The alterations are indexed (#1 - #5) in chromosomal order. The focal high-level amplification (#2) and homozygous deletion (#5) are nested within the larger single copy gain (#1) and loss (#4) **(D) **The observed alterations identified by rSW-seq were classified according to their expected copy numbers and compared with the 5 predefined alterations.

To test this, we simulated 1 Mb single copy gains (3 copies) containing a smaller (50 kb, 100 kb, 200 kb, and 300 kb) two copy gain (4 copies) in 100 Mb artificial chromosomes. The alteration found in the first scan was used as template for the second scan of the algorithm. The performance of the second scan in identifying the implanted two copy gains was measured with different *t_gain _*levels (Figure 6A). The 100 kb two copy gains were identified at >80% sensitivity and >80% precision at *t_gain _*0.06. The smaller copy number ratio (4 vs 3 copies) is responsible for the smaller *t_gain _*compared to the threshold level required for detecting single copy gain (3 copies vs 2). Focal homozygous deletions (zero copy) nested in single copy number losses (1 copy) were also simulated and tested for the performance (Figure 6B). In this case, the decrease in sensitivity level was not observed with higher *t_loss _*level, possibly due to the absence of tumor reads in the homozygous deletion. The use of *t_loss _*0.16 was able to detect all tested sizes of homozygous deletions with >90% sensitivity and >90% precision.

We also simulated a set of complex alterations that contain 2 single copy gains (3 copies; 1 Mb) and a single copy loss (1 copy; 500 kb) as well as 1 high-level amplification (4 copies; 100 kb) and homozygous deletion (zero copy; 100 kb) in a single profile (Figure 6C). rSW-seq was able to identify focal high-level amplification and homozygous deletion separately from their nested larger single copy gain and loss. We also note that a small region with no copy number change that separates large single copy gain can be identified as an isolated alteration, e.g., single copy loss with respect to single copy gain. The observed alterations found in 100 recursive tests were compared with the simulated alterations with the matched copy numbers to measure the performance of rSW-seq (Figure 6D). Not surprisingly, it shows that the performance of rSW-seq at identifying multilayered alterations is highly influenced by the copy number differences in the nested alterations, *e.g*., relatively poor performance for high-level amplification (4 copies) nested in single copy gain (3 copies).

### The performance of rSW-seq in real sequencing data

To test the performance of the algorithm in real sequencing data, we applied rSW-seq to the sequencing data initially analyzed by SegSeq [15]. This dataset contained three pairs of cancer- derived cell lines (tumor and matched normal), each of which was comprised of 25 - 35 million reads. The dataset also includes genomic profiles generated on the same samples using high-resolution array-CGH platform (Affymetrix genomewide SNP 6.0) that can be used for comparison. rSW-seq was applied using the *t *levels determined in simulation tests and a score cutoff of 100. Then we compared the results of rSW-seq with those of SegSeq for segments corresponding to copy number gains (read ratio >1.5) and losses (read ratio <0.5) in each of the three cell lines for the total of six comparisons (Table 1). We found a high level of concordance (79.7% - 98.6%) between the segmentation results of rSW-seq and SegSeq, where the concordance was defined as the fraction of overlapping region identified by the two methods over the total segments size found in either method. When the results are compared with independent segmentation results obtained from Affymetrix array-CGH, rSW-seq showed higher concordance rates as compared with SegSeq in 5 out of 6 comparisons.

**Table 1:** Comparison of overlap between alterations.

	Ratio	Method	HCC1143	HCC1954	H2347
Concordance rate (%)^a^	>1.5	rSW-seq	94.2	90.9	97.4
					
		SegSeq	83.6	79.7	99.5
	
	<0.5	rSW-seq	93.0	97.1	98.6
		SegSeq	91.0	93.2	93.8

Concordance rate vs array-based profile (%)^b^	>1.5	rSW-seq	96.8	96.4	70.0
					
		SegSeq	95.4	94.6	26.3
	
	<0.5	rSW-seq	83.9	57.2	42.4
		SegSeq	76.9	57.8	33.5

^a^Concordance rate was measured between the results of rSW-seq and SegSeq, and is defined as (overlapping region between two methods in bp)/(total regions identified by either rSW-seq or Segseq in bp). ^b^Concordance rate is (overlapping region between array-based and sequencing based in bp)/(regions identified by array-based or sequencing-based in bp).

**Figure 7:**
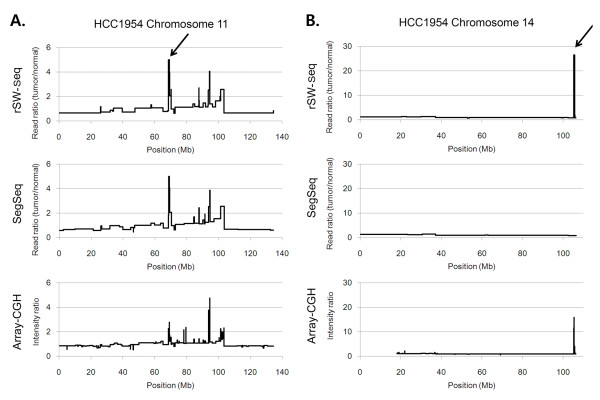
**The comparison of three segmentation profiles**. **(A) **The segmentation results for chromosome 11 in HCC1954 are shown for the two sequencing-based methods, rSW-seq (top) and SegSeq (middle), and for an array-based method (bottom). The profiles are very similar in this case. The arrow indicates a high-level amplification peak located at 11q13, where the array-based profile gives a reduced signal. **(B) **Three plots of chromosome 14 are also shown for the same cell line. The arrow indicates the high-level amplification at 14q32, which is observed in the rSW-seq and array-based profiles.

The individual chromosomal profiles obtained by rSW-seq and SegSeq are notably similar (see Additional file 1: Figure S3). For example, in chromosome 11 in the tumor cell line HCC1954, two methods show similar profiles overall, which is also consistent with array-CGH results (Figure 7A). A focal amplification residing at ~70 Mb of chromosome 11 (11q13) contains well-known cancer genes *FGF3, FGF4 *and *CCND1 *and appears as a dominant peak in read ratios both for rSW-seq and SegSeq as compared to the hybridization-based intensity ratio. Such is indicative of the higher dynamic range of the sequencing-based measures, as previously shown for *ERBB2 *amplification in the same dataset HCC1954 [15]. For the 4 high-level amplifications by SegSeq showing read ratio >8 (5p15, 8q23 and 17q12 on HCC1954 and 19p13 on HCC1143), all were recovered by rSW-seq. There are some differences in the two profiles as well. One is a high-level amplification identified by rSW-seq on 14q32 in HCC1954 (Figure 7B). This amplification is supported by the array-CGH profile and it contains loci for breast cancer-related signaling molecule *AKT1 *[20] in this breast cancer cell line. With respect to candidates for homozygous deletions, three loci in H2347 were coincident between rSW-seq and SegSeq (6q24, 9p23 and 17p12). But rSW-seq also identified 5 additional candidates for homozygous deletions in HCC-1143, which include cancer-related genes such as *TRAPPC6B *(14q21), *AML1 *and *RUNX1 *(both on 21q22), worthy of further investigation.

It should be noted that our simulation tests above are based on idealized copy number ratios for CNAs, e.g., 3/2 of tumor and normal read ratio for single copy gain. Considering the tissue heterogeneity in tumors, this is unlikely to be true in actual data. It is possible that the methods used here for cell line-derived data may require additional optimization for analysis of sequencing data from primary cancer cells.

## Conclusions

We have proposed rSW-seq as an iterative method that can be used to discover CNAs efficiently, including those in a complex configuration. Among the methods for single-end read-based copy number analysis [11,12,18], SegSeq and rSW-seq are similar in that they are designed to make CNA calls by direct comparison of tumor and paired normal genomes [15]. One key difference, however, is that SegSeq first identifies the potential breakpoints (point-centric) and merges neighboring windows to obtain candidate segments, while rSW-seq directly captures potential CNAs as regions with substantial bias in tumor vs normal reads counts (region-centric). Global algorithms such as rSW-seq are more likely to perform better at detecting larger or more subtle CNAs, for which point-centric algorithms might miss boundaries that do not show clear differences in read density. In our simulation, rSW-seq showed improved performance compared to SegSeq (e.g., better precision at comparable sensitivity level, Figure 5). An important advantage of rSW-seq also is that a window size, which can change the results substantially for SegSeq, does not need to be specified. However, the performance of the algorithms in real datasets remains to be studied more extensively. Most likely, these methods should complement each other in making reliable calls for candidate CNAs. When the data consists of paired-end reads (PEM), the algorithms [21,22] designed for such data should also provide complementary information.

As next-generation sequencing becomes more widely available, more whole-genome sequencing data will be generated for cancer studies. rSW-seq provides a solution for effective screening of cancer-specific CNAs for better understanding of the tumor biology and discovery of biomarkers.

## Methods

### Details of the algorithm

Given *N_T _*and *N_N _*as the total number of tumor and normal reads in the dataset, respectively, the copy number gain-detection algorithm is presented in the following pseudocode.

1 *W_T _*= 2 × *N_N_*/(*N_T _*+ *N_N_*), *W_N _*= -2 × *N_T_*/(*N_T _*+ *N_N_*)

2 *k *= 1

3 Repeat

4    *S *= 0,* l *= 1,* S_max _= 0*

5    For *i *in 1 to *N_T _*+ *N_N_*

6       if *r_i _*is tumor and unmasked then *S *= *S *+ *W_T_*

7       if *r_i _*is normal and unmasked then *S *= *S *+ *W_N_*

8       if *S *>*S_max _*then *S_max _*= *S*, *l_max _*= *l, m_max _*= *i*

9       if *S *< 0 then *S* = 0,* l *= *i *+ 1

10    End For

11 Report *S_max_, l_max_, m_max_*

12 Mask *r_i _*from *l_max _*to *m_max_*

13 *k *= *k *+ 1

14 Until *S_max _*= 0

Each chromosome scan produces a single CNA candidate, and the scanning iterates until no more positive-scoring segments can be found. The reads corresponding to the identified CNAs are masked before the iteration continues. The computational complexity is 0(*k *× (*N_T _*+ *N_N_*)) When *k *represents the number of CNAs detected. It is of note that the *N_T _*and *N_N _*are the read number of entire dataset each from tumor and matched normal sequencing dataset.

In the following we show that the segment $\left[s_{l_{max}},s_{m_{max}}\right]$ identified in the above pseudocode is the maximum segment. Note that the segment $\left[s_{l_{max}},s_{m_{max}}\right]$ is the same as $\left[S_{l_{k_{0}}},S_{m_{k_{0}}}\right]$ in the main text. We will use $\left[S_{l_{k_{0}}},S_{m_{k_{0}}}\right]$ to refer to the segment identified in the above pseudocode.

#### Proposition

The segment $\left[S_{l_{k_{0}}},S_{m_{k_{0}}}\right]$ is the maximum segment.

Proof. Assume that [*s_l_, s_m_*] is the segment that maximizes the partial cumulative sum $S(l,m)=\sum_{j=l}^{m}W_{j}$ Without loss of generality, we assume *S*(*l, m*) > 0 (otherwise, there will be no tumor read and no copy gain region will be identified).

Remember that *K *is the integer such that *S*(*l_k_, i*) > 0 for all *i *≥ *l_k_*. We first prove the following remark.

#### Remark

For each *k *= 1,...,*K*, *S*(*l_k_, m_k_*) = max{*S*(*i, j*),*l_k _*≤ *i *≤ *j *<*l*_*k*+1_}.

Case1: *k *= *K*. For any *l_k _*≤ *i *≤ *j *<*l_k _*+ 1 We have *S*(*l_k_, j*) = *S*(*l_k_*, i - 1) + *S*(*i, j*) (define *S*(*l_k_, l_k_*-1) as 0). By the definition of *k *we have *S*(*l_k_, l_k_*-1) ≥ 0 Thus, *S*(*l_k_, m_k_*) ≥ *S*(*l_k_, j*) ≥ *S*(*i, j*) and henc *S*(*l_k_, m_k_*) = max{*S*(*i, j*),*l_k _*≤ *i *≤ *j *<*l*_*k*+1_}

Case 2: *k *<*K*. If *l_k _*= *l*_*k*+1 _-1, the remark holds immediately. Assume *l_k _*<*l*_*k*+1 _- 1 we then have *S*(*l_k_*, i) > 0 for all *l_k _*≤ *i *≤ *l*_*k*+1 _-2, since *l*_*k*+1 _-1 is the first index after *l_k _*such that *S*(*l_k_, i*) < 0 (*i *≥ *lk*). Suppose that *S*(*i*_0_,*j*_0_) = max{*S*(*i, j*), *l_k _*≤ *i *≤ *j *<*l*_*k*+1_} We have, *S*(*l_k_, i*_0_) = *S*(*l_k_, i*_0 _- 1) + *S*(*i*_0_,*j*_0_) ≥ *S*(*i*_0_,*j*_0_), where the equality holds if and only if *l_k _*= *i*_0_. Thus, we get *l_k _*= *i*_0_. On the other hand, since *S*(*l_k_, m_k_*) ≥ *S*(*l_k_, i*) for all *l_k _*≤ *i *<*l*_*k*+1_, we have *S*(*l_k_, m_k_*) ≥ *S*(*l_k_, j*_0_) = S(*i*_0_,*j*_0_) and *S*(*l_k_, m_k_*) = max{*S*(*i, j*), *l_k _*≤ *i *≤ *j *<*l*_*k*+1_}.

Now we prove $\left[S_{l_{k_{0}}},S_{m_{k_{0}}}\right]$ is the maximum segment [*s_l_, s_m_*]. Let 1 ≤ *k*_1 _≤ *k*_2 _≤ *K *be the integer such that $l_{k_{1}}\leq l<l_{k_{1}+1}$ and $l_{k_{2}}\leq m<l_{k_{2}+1}$.

If *k*_1 _= *k*_2_, we have $l_{k_{1}}\leq l\leq m<l_{k_{1}+1}$. According to the above remork, we have$S(l_{k_{1}},m_{k_{1}})\geq S(l,m)\geq S(l_{k_{0}},m_{k_{0}})\geq S(l_{k_{1}},m_{k_{1}})$ and hence $\left[S_{l_{k_{0}}},S_{m_{k_{0}}}\right]$ is the maximum segment.

If *k*_1 _<*k*_2_, we have $l_{k_{1}}\leq l<l_{k_{1}+1}\leq m<l_{k_{2}+1}$. If $l=l_{k_{1}+1}-1$, we have *S*(*l, l*) < 0.

However, *S*(*l, m*) = *S*(*l, l*) + *S*(*l *+ 1, *m*) <*S*(*l *+ 1, *m*), which contradicts the fact that [*s_l_, s_m_*] is the maximum segments. Hence, we have $l_{k}\leq l<l_{k_{1}+1}-1$. Thus, $S(l_{k_{1}},l-1)\geq 0$. Since $S(l_{k_{1}}l_{k_{1}+1}-1)=S(l_{k_{1}},l-1)+S(l,l_{k_{1}+1}-1)<0$, we have $S(l,l_{k_{1}+1}-1)<0$. Thus, $S(l,m)=S(l,l_{k_{1}+1}-1)+S(l_{k_{1}+1},m)<S(l_{k_{1}+1},m)$, which again contradict the fact that [*s_l_, s_m_*] is the maximum segments. Hence the proposition was proved.

### Simulation tests

We simulated 100 Mb artificial chromosomes that contain a million virtual tags split equally between tumor (T) and normal (N) reads. To simulate normal reads, we randomly placed a half million tags across the chromosome. Tumor reads were positioned to simulate tumor-specific single copy gain (3 copies) and loss (1 copy) with respect to the normal genome (2 copies). To simulate a 1 Mb single copy gain, for example, we randomly assigned the positions of a half million tags across 100.5 Mb chromosome. Then, the tags corresponding to the additional 0.5 Mb segment were moved to a predefined 1 Mb segment within the chromosome to simulate single copy gain. For single copy loss, one half of tumor reads were randomly positioned but excluded in a predefined segment corresponding to single copy loss, while the other half of tumor reads were positioned across the chromosome. Alterations identified by the algorithm were compared with the predefined alterations by the extent of overlap (true positive, TP). The means of false negative (FN) and positive (FP) rates were also calculated for 100 artificial chromosomes to measure sensitivity (%; TP/TP + FN) and precision (%; TP/TP + FP).

To measure the accuracy of boundary mapping, the separating distance (bp) between the boundaries of estimated and predefined alterations were measured. In case of multiple alterations in a single chromosome, the most left- and right-ward boundaries were selected as start and end points of observed alterations. The differences in the boundary mappings were measured separately for the observed boundaries that reside in- or outside the predefined alterations. We also tested the robustness of algorithm in scanning orientation using the same set of artificial chromosomes. For each of observed alteration found in the left-to-right orientation, we checked whether the same alteration was identified by the reverse (right-to-left) scanning. The effect of subsequent score and significance-based threshold was assessed by filtering out the observed alterations using 11 scales of SW scores (50 to 150) or significance-cutoffs (10^-5 ^to 10^-15^). The performance testing at different coverage level was performed by placing 3 million and 10 million virtual tags on 100 Mb artificial chromosomes.

To measure the significance level of observed alterations, we counted the number of tumor and normal reads within the alteration and used a statistical method previously described for differential analysis of sequencing tags [19]. According to this model, the probability of observing *t *tumor reads in a defined segment containing *n *normal reads can be calculated assuming random distribution of sequencing reads and given *N_T _*and *N_N_*. For copy number losses (*t/n *<*N_T_*/*N_N_*), the probability of observing less than or equal to *t *number of tumor reads with *n *normal reads is the following [19]:

$$P(t|n,N_{T,}N_{N})={\sum_{i=0}^{t}\left(\frac{N_{T}}{N_{N}}\right)}^{{}^{i}}\frac{(i+n)!}{i!n!{\left(1+\frac{N_{T}}{N_{N}}\right)}^{\left(i+n+1\right)}}$$

For copy number gain (*t*/*n *>*N_T_*/*N_N_*), the probability of observing equal to or more than *t *tumor reads is 1 - *P*(*t *- 1|*n*).

For the second set of simulations based on a test chromosome from a real genome, we used the human chromosome 1 (~250 Mb) as a template. To simulate copy number changes, we used a strategy described previously [18]. First, we randomly selected two chromosomal positions ('source' and 'target') and the sequence of defined size (10 kb - 1 Mb; 8 scales) at the source position was copied into the target position. This results in copy number gain and loss at the source and target positions, respectively. For an individual test chromosome, 16 alterations (8 copy gains and 8 copy losses in different sizes) were simulated at random positions. The test chromosome was further concatenated to an unmodified template sequence, making the simulated alterations correspond to single copy gains and losses. To account for mappability, 36 bp reads were randomly selected from simulated chromosomal sequence and mapped using Bowtie [23], keeping only uniquely mapped reads. Sequencing depths of 1 million to 20 million reads were tested. In case of rSW-seq, we used the optimized setting (SW-score of 80 and threshold level of *t_gain_*= 0.1 and *t_loss _*= 0.16). For SegSeq, we used default parameter setting except for the window size *w *(*w *= 400 for 1 - 5 million reads and *w *= 1000 for 10 - 20 million reads) since the use of default parameter (*w *= 400) at higher coverage showed poor performance (<80% of sensitivity for single copy gains <500 kb).

### Datasets

Sequencing data for the three cell line pairs of tumor and matched normal genomes (HCC1954, HCC1143 and H2347) were downloaded from accompanying website for SeqSeq [15]. For the comparison of the results obtained by rSW-seq, we used the segmentation results of the same datasets analyzed with SegSeq at its default setting. The profiles of the same cell line pairs obtained from the Affymetrix SNP 6.0 platforms were also downloaded from the same website. We calculated the log_2 _ratios of the signal intensities form tumor and paired normal lines and performed the segmentation using CBS algorithm [17].

### Code availability

Available upon request.

## Authors' contributions

TMK designed the algorithm. LJL and RX helped with refinement and implementation of the algorithm. PJP supervised the project. TMK, RX and PJP wrote the manuscript. All have read the manuscript and approved the final version.

## Supplementary Material

Additional file 1 **Supplementary Figures**. **Figure S1**: Effect of filtering by score and significance thresholds for gains. **Figure S2: **Effect of filtering by score and significance thresholds for losses. **Figure S3: **Comparison of chromosomal profiles. Click here for file

## References

[B1] Frohling S Dohner H Chromosomal abnormalities in cancer N Engl J Med 2008 359 722 734 10.1056/NEJMra0803109 18703475

[B2] Albertson DG Collins C McCormick F Gray JW Chromosome aberrations in solid tumors Nat Genet 2003 34 369 376 10.1038/ng1215 12923544

[B3] Pinkel D Albertson DG Array comparative genomic hybridization and its applications in cancer Nat Genet 2005 37 Suppl S11 S17 10.1038/ng1569 15920524

[B4] Albertson DG Pinkel D Genomic microarrays in human genetic disease and cancer Hum Mol Genet 2003 12 Spec No 2 R145 R152 10.1093/hmg/ddg261 12915456

[B5] Snijders AM Nowak N Segraves R Blackwood S Brown N Conroy J Hamilton G Hindle AK Huey B Kimura K Assembly of microarrays for genome-wide measurement of DNA copy number Nat Genet 2001 29 263 264 10.1038/ng754 11687795

[B6] Wang TL Maierhofer C Speicher MR Lengauer C Vogelstein B Kinzler KW Velculescu VE Digital karyotyping Proc Natl Acad Sci USA 2002 99 16156 16161 10.1073/pnas.202610899 12461184 PMC138581

[B7] Bentley DR Whole-genome re-sequencing Curr Opin Genet Dev 2006 16 545 552 10.1016/j.gde.2006.10.009 17055251

[B8] Margulies M Egholm M Altman WE Attiya S Bader JS Bemben LA Berka J Braverman MS Chen YJ Chen Z Genome sequencing in microfabricated high-density picolitre reactors Nature 2005 437 376 380 16056220 10.1038/nature03959 PMC1464427

[B9] Mardis ER The impact of next-generation sequencing technology on genetics Trends Genet 2008 24 133 141 18262675 10.1016/j.tig.2007.12.007 PMC2680276

[B10] Morozova O Marra MA Applications of next-generation sequencing technologies in functional genomics Genomics 2008 92 255 264 10.1016/j.ygeno.2008.07.001 18703132

[B11] Alkan C Kidd JM Marques-Bonet T Aksay G Antonacci F Hormozdiari F Kitzman JO Baker C Malig M Mutlu O Personalized copy number and segmental duplication maps using next-generation sequencing Nat Genet 2009 41 1061 1067 10.1038/ng.437 19718026 PMC2875196

[B12] Yoon S Xuan Z Makarov V Ye K Sebat J Sensitive and accurate detection of copy number variants using read depth of coverage Genome Res 2009 19 1586 1592 10.1101/gr.092981.109 19657104 PMC2752127

[B13] Smith TF Waterman MS Identification of common molecular subsequences J Mol Biol 1981 147 195 197 10.1016/0022-2836(81)90087-5 7265238

[B14] Price TS Regan R Mott R Hedman A Honey B Daniels RJ Smith L Greenfield A Tiganescu A Buckle V SW-ARRAY: a dynamic programming solution for the identification of copy-number changes in genomic DNA using array comparative genome hybridization data Nucleic Acids Res 2005 33 3455 3464 10.1093/nar/gki643 15961730 PMC1151590

[B15] Chiang DY Getz G Jaffe DB O'Kelly MJ Zhao X Carter SL Russ C Nusbaum C Meyerson M Lander ES High-resolution mapping of copy-number alterations with massively parallel sequencing Nat Methods 2009 6 99 103 10.1038/nmeth.1276 19043412 PMC2630795

[B16] Lai WR Johnson MD Kucherlapati R Park PJ Comparative analysis of algorithms for identifying amplifications and deletions in array CGH data Bioinformatics 2005 21 3763 3770 10.1093/bioinformatics/bti611 16081473 PMC2819184

[B17] Olshen AB Venkatraman ES Lucito R Wigler M Circular binary segmentation for the analysis of array-based DNA copy number data Biostatistics 2004 5 557 572 10.1093/biostatistics/kxh008 15475419

[B18] Xie C Tammi MT CNV-seq, a new method to detect copy number variation using high-throughput sequencing BMC Bioinformatics 2009 10 80 10.1186/1471-2105-10-80 19267900 PMC2667514

[B19] Audic S Claverie JM The significance of digital gene expression profiles Genome Res 1997 7 986 995 9331369 10.1101/gr.7.10.986

[B20] Zinda MJ Johnson MA Paul JD Horn C Konicek BW Lu ZH Sandusky G Thomas JE Neubauer BL Lai MT AKT-1, -2, and -3 are expressed in both normal and tumor tissues of the lung, breast, prostate, and colon Clin Cancer Res 2001 7 2475 2479 11489829

[B21] Hormozdiari F Alkan C Eichler EE Sahinalp SC Combinatorial algorithms for structural variation detection in high-throughput sequenced genomes Genome Res 2009 19 1270 1278 10.1101/gr.088633.108 19447966 PMC2704429

[B22] Lee S Hormozdiari F Alkan C Brudno M MoDIL: detecting small indels from clone-end sequencing with mixtures of distributions Nat Methods 2009 6 473 474 10.1038/nmeth.f.256 19483690

[B23] Langmead B Trapnell C Pop M Salzberg SL Ultrafast and memory-efficient alignment of short DNA sequences to the human genome Genome Biol 2009 10 R25 10.1186/gb-2009-10-3-r25 19261174 PMC2690996

