# Supplementary material for: rSW-seq: Algorithm for detection of copy number alterations in deep sequencing data
Source: BMC Bioinformatics. 2010 Aug 18;11:432. doi: 10.1186/1471-2105-11-432 (PMC2939611; doi:10.1186/1471-2105-11-432)
Supplement: Additional file 1 — Supplementary Figures. Figure S1: Effect of filtering by score and significance thresholds for gains. Figure S2: Effect of filtering by score and significance thresholds for losses. Figure S3: Comparison of chromosomal profiles. [file 1471-2105-11-432-S1.PDF]

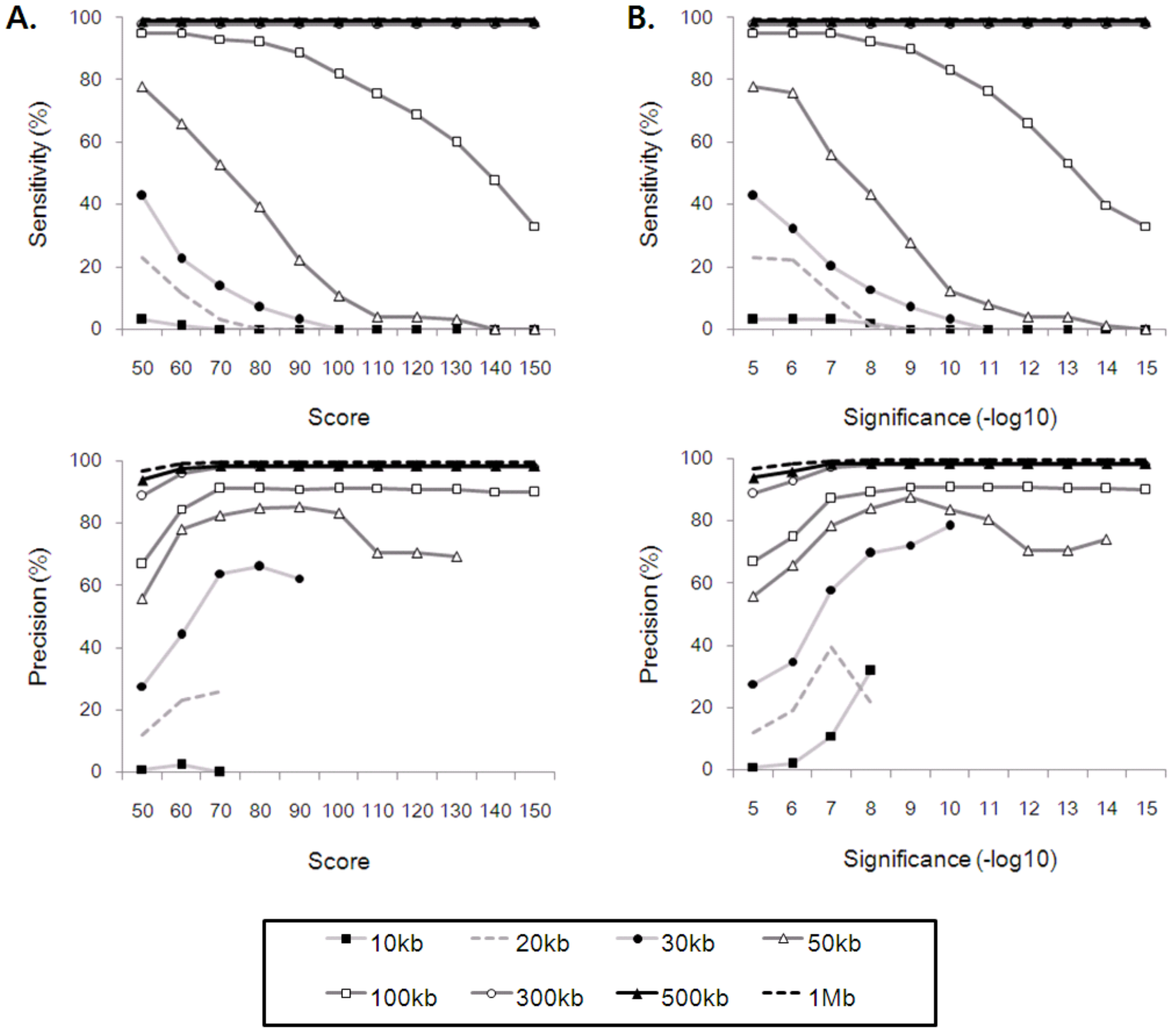

Supplementary Figure 1 Effect of filtering by score and significance thresholds for gains. (A) The sensitivity and precision in detecting single copy number gain was measured using SW-score cutoffs (50 to 150) and t_{gain} = 0.1. **(B)** Same quantities were measured when filtered by significance cutoffs (10^{-5} to 10^{-15}).

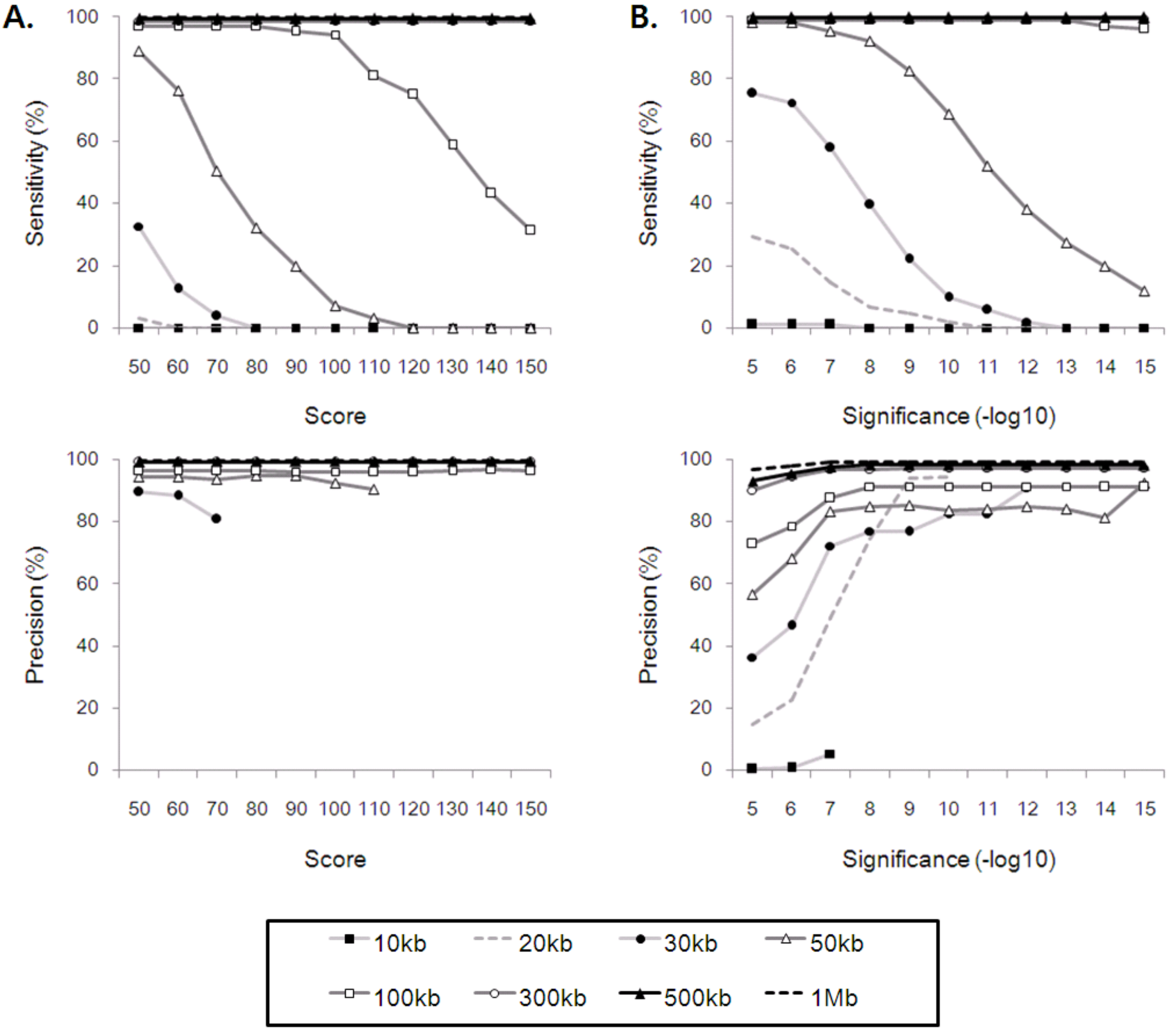

Supplementary Figure 2. Effect of filtering by score and significance thresholds for losses. Same as Supplementary Figure 1 but for losses.

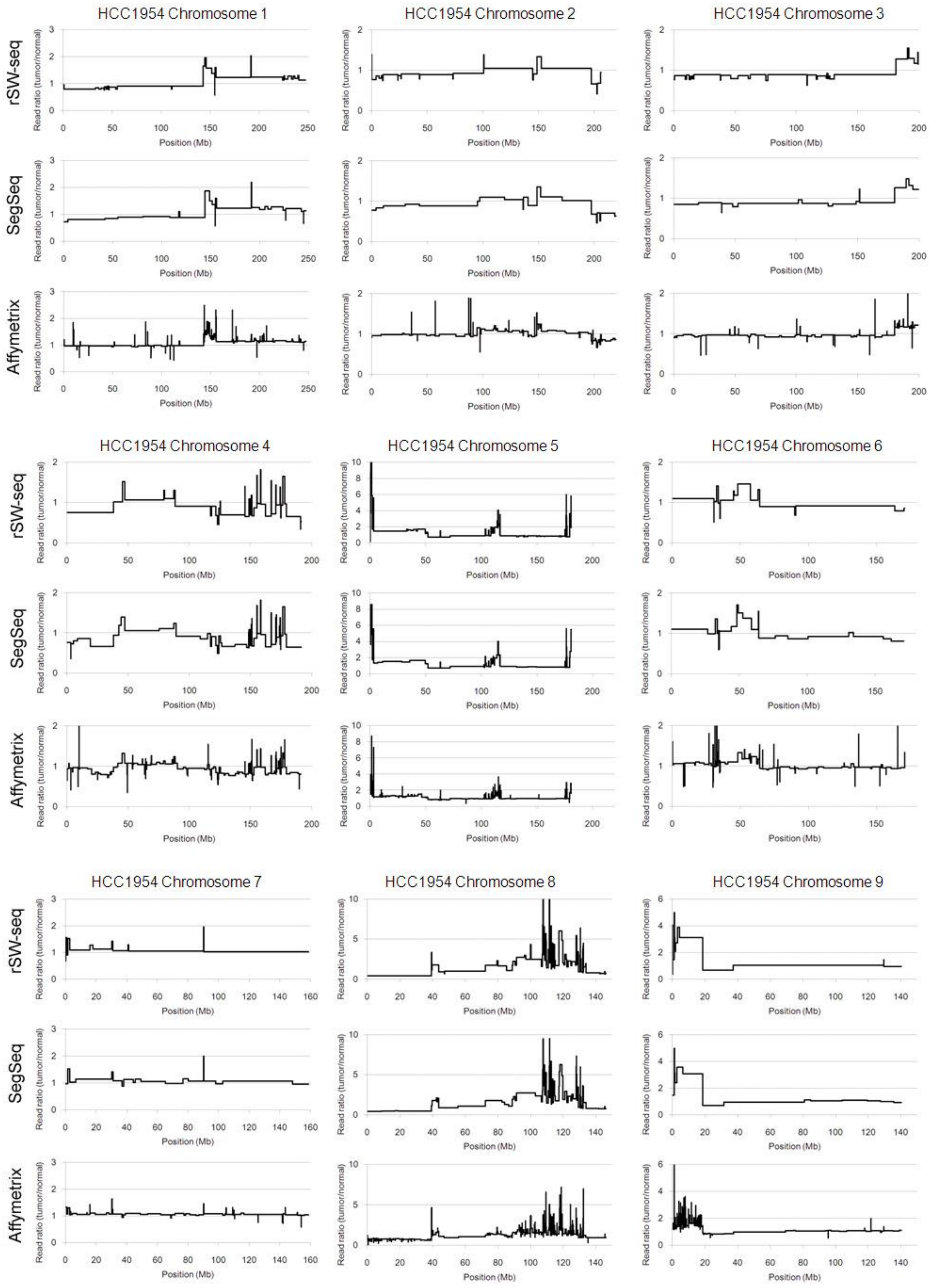

Supplementary Figure 3. Comparison of chromosomal profiles. For 3 cell line tested (HCC1954, HCC1143 and H2347), the segmentation results by rSW-seq are shown for individual chromosomes. For comparison, SegSeq results for the same data and independent Affymetrix SNP 6.0 profiles are also shown.

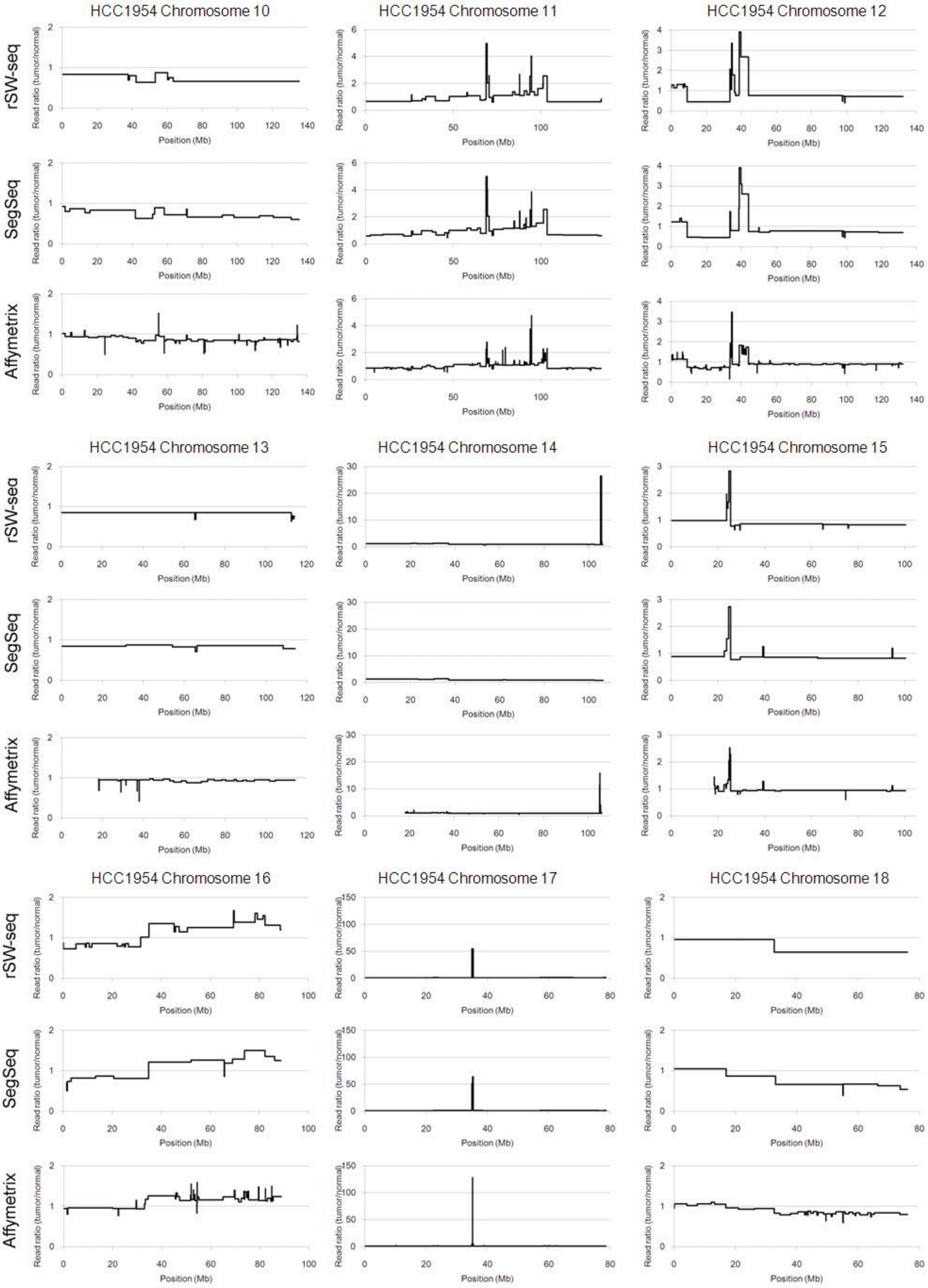

Supplementary Figure 3. Comparison of chromosomal profiles. (Continued)

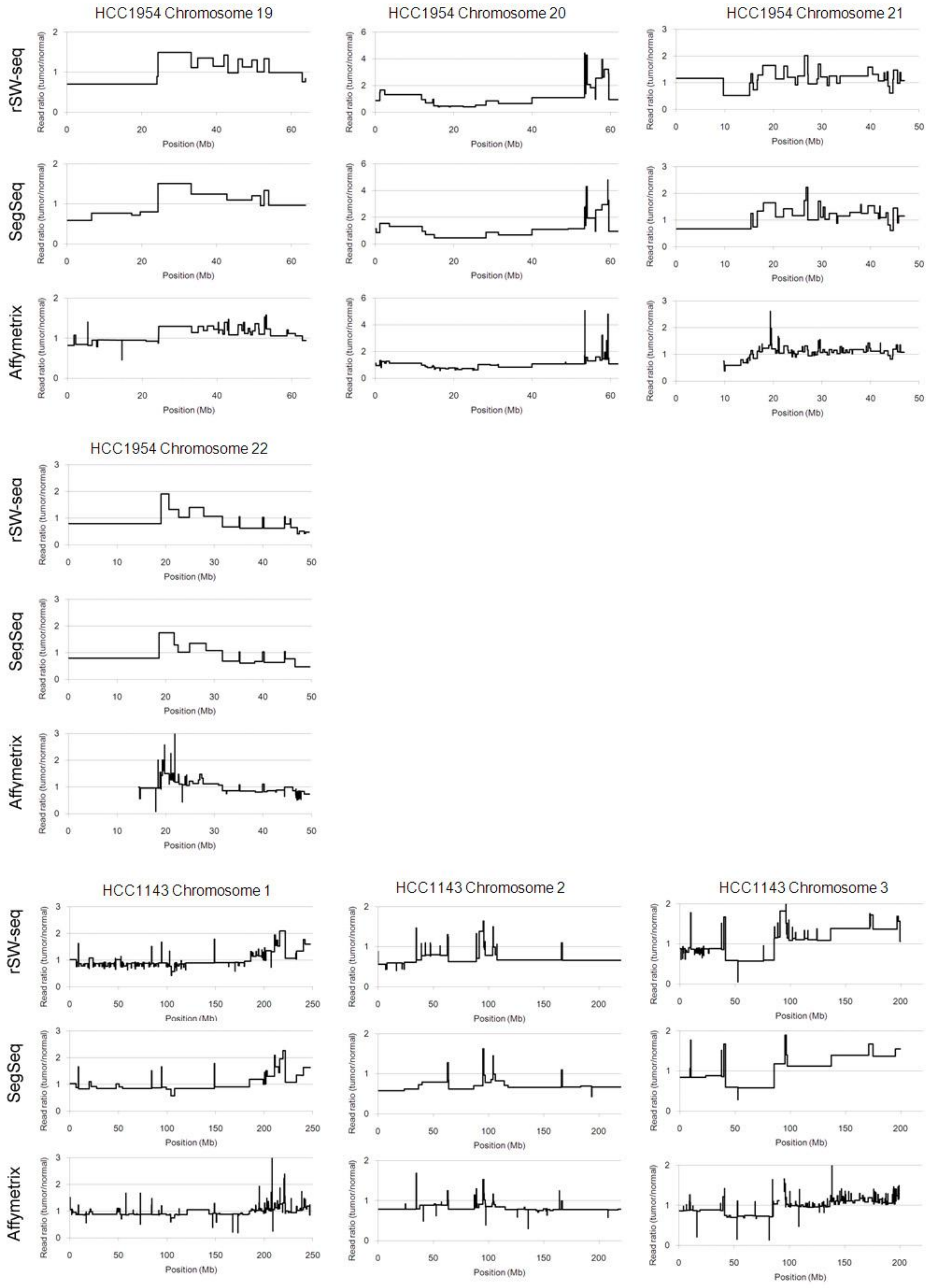

Supplementary Figure 3. Comparison of chromosomal profiles. (Continued)

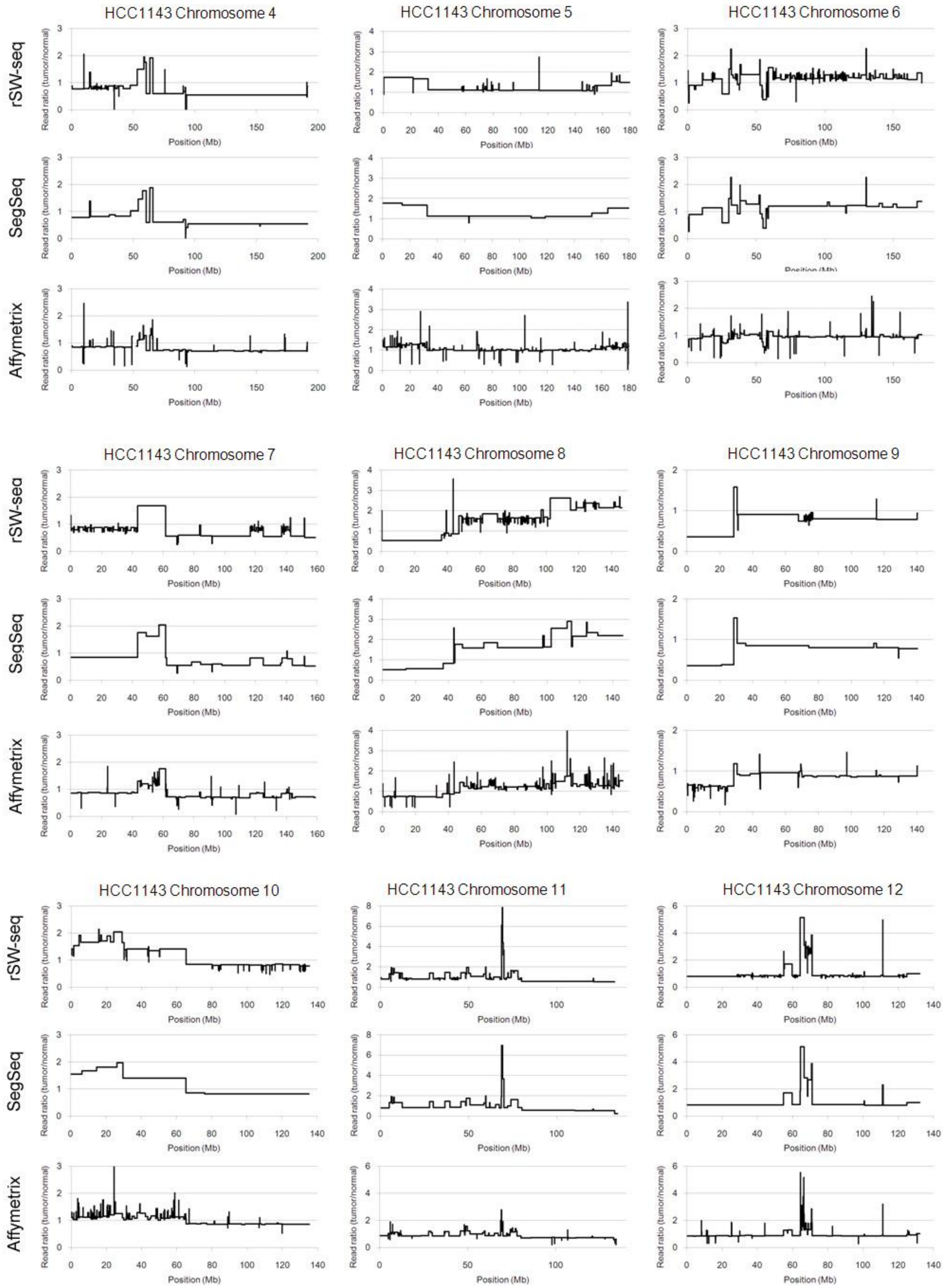

Supplementary Figure 3. Comparison of chromosomal profiles. (Continued)

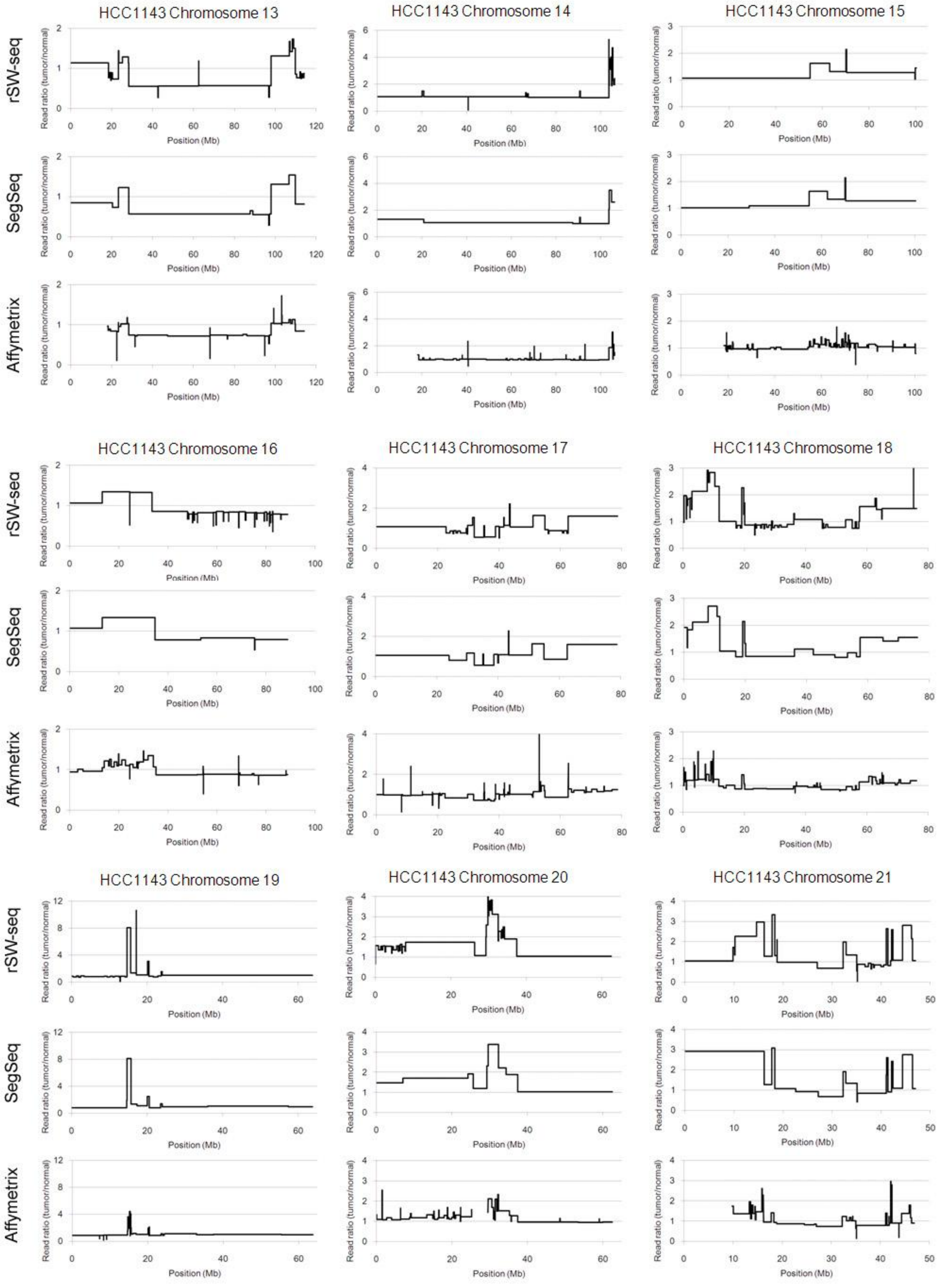

Supplementary Figure 3. Comparison of chromosomal profiles. (Continued)

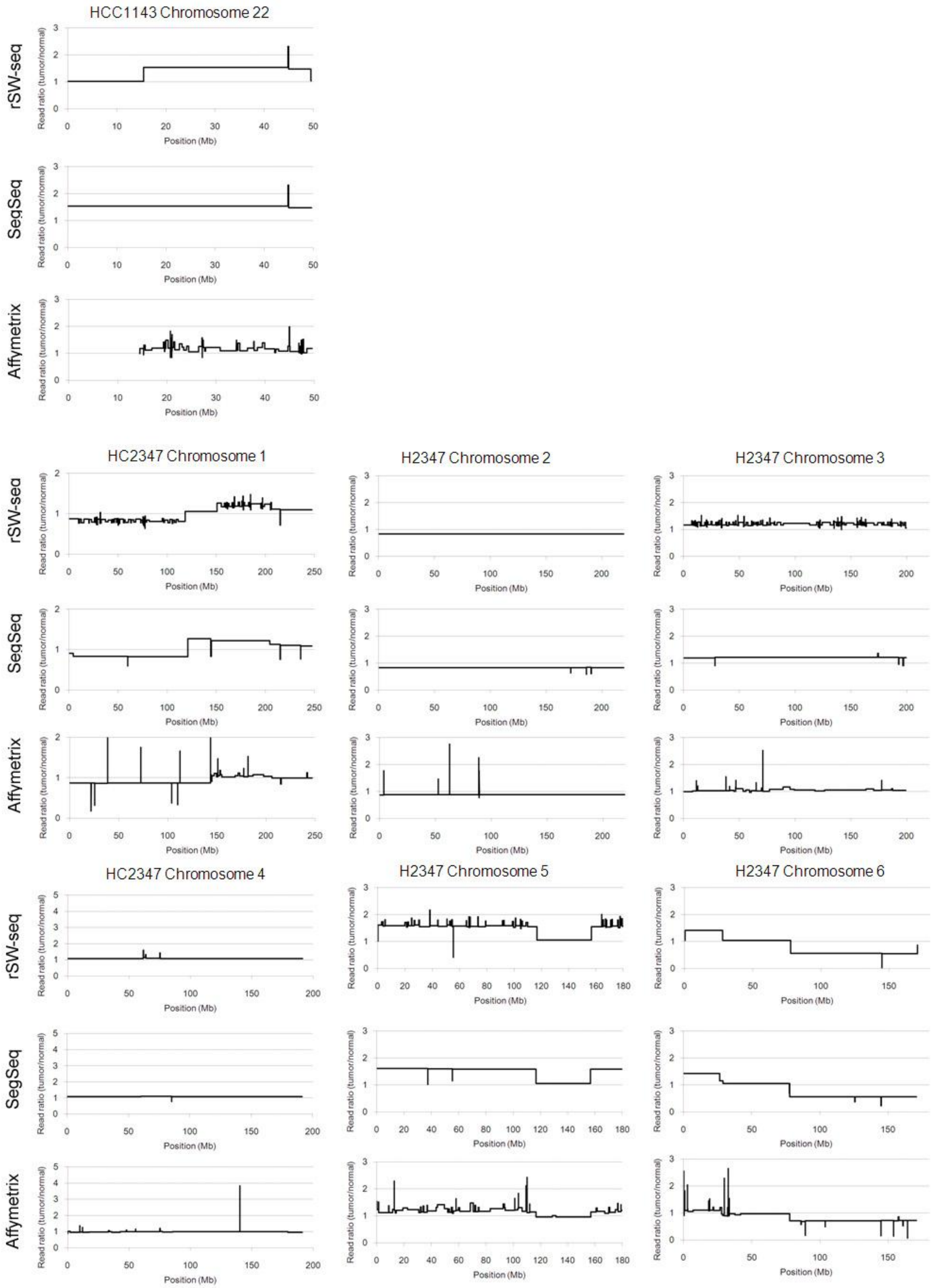

Supplementary Figure 3. Comparison of chromosomal profiles. (Continued)

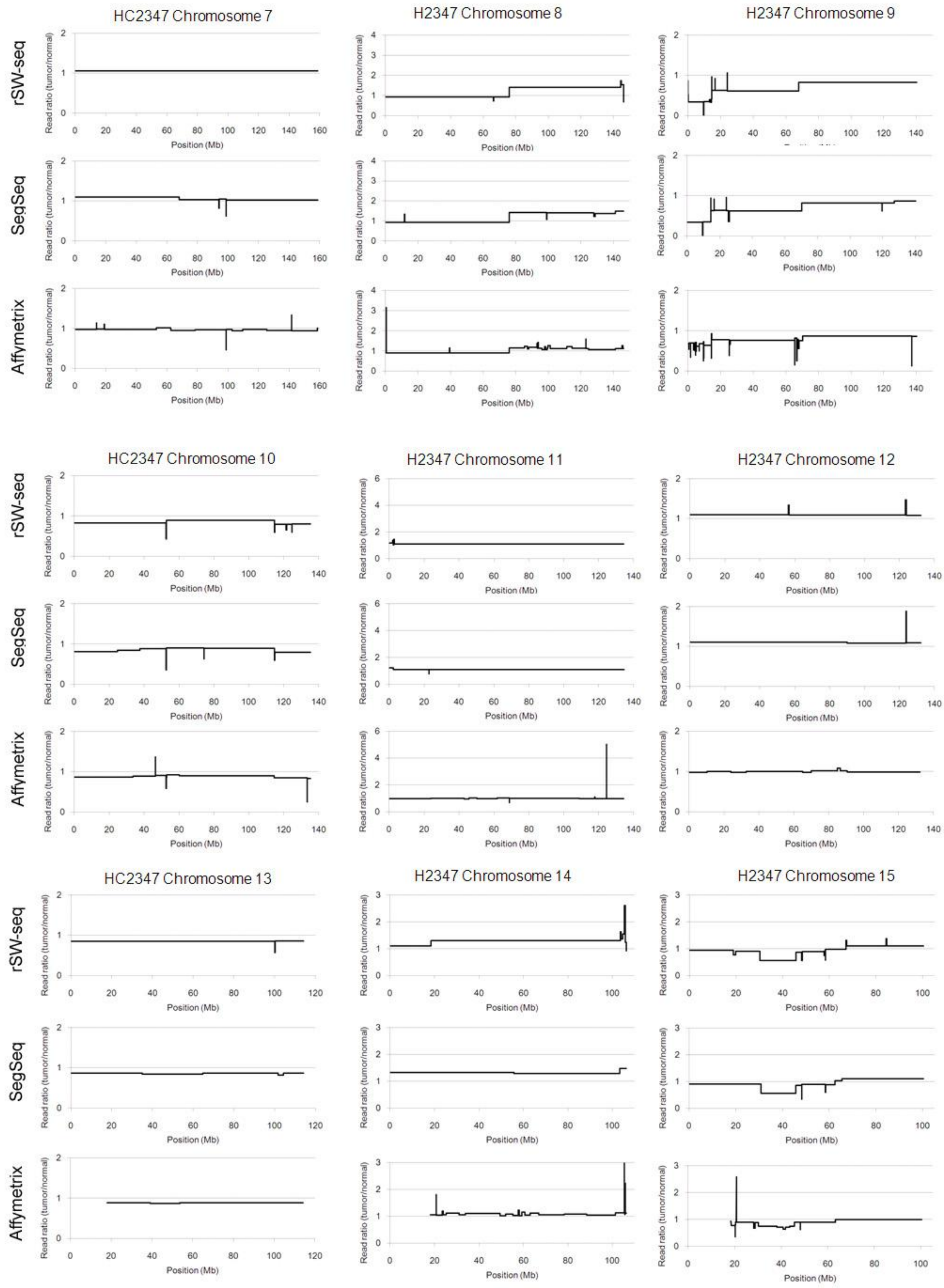

Supplementary Figure 3. Comparison of chromosomal profiles. (Continued)

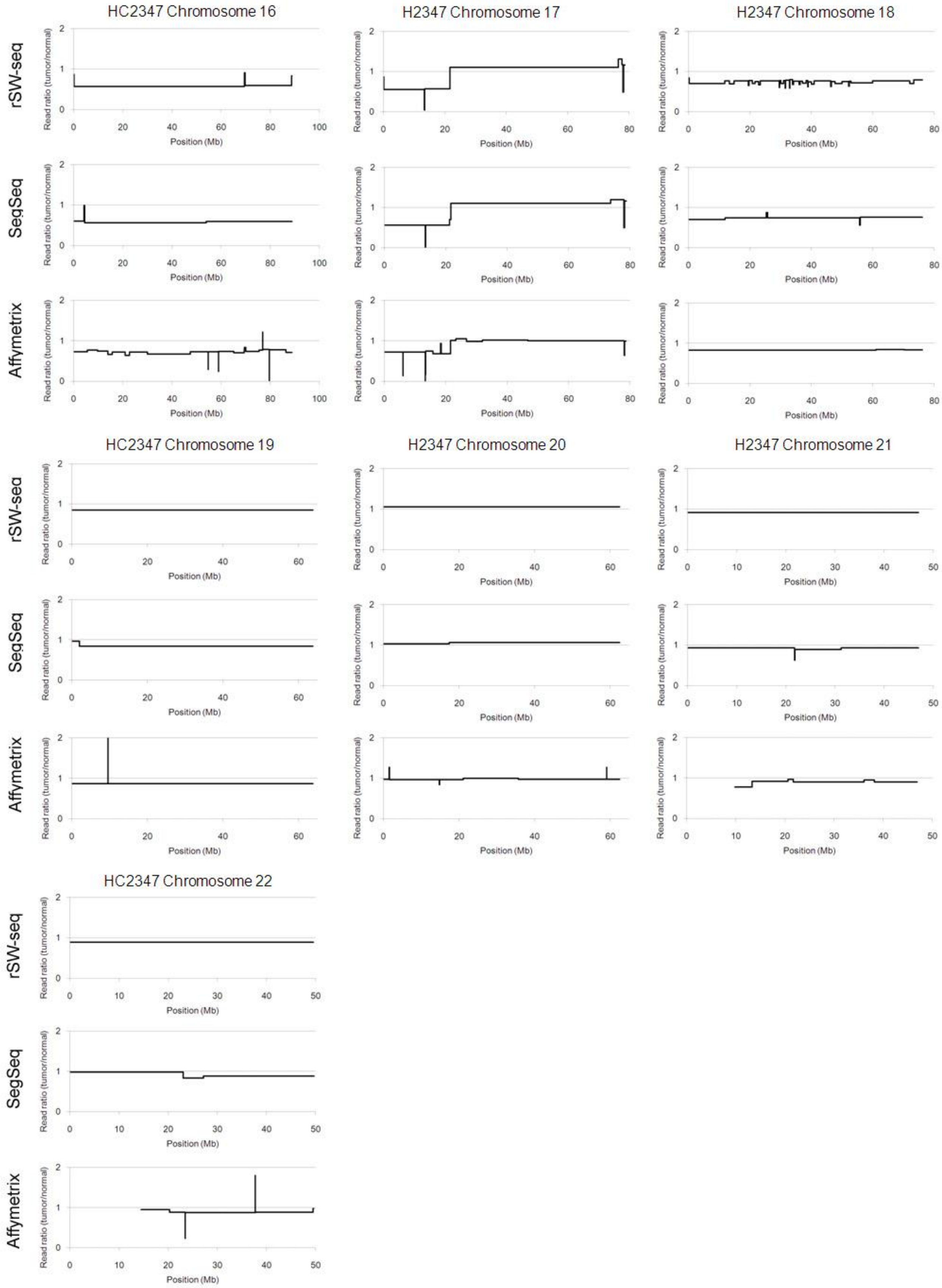

Supplementary Figure 3. Comparison of chromosomal profiles. (Continued)
